# VISIT-TS: A multimedia tool for population studies on tic disorders

**DOI:** 10.12688/f1000research.7196.2

**Published:** 2016-10-07

**Authors:** M. Jonathan Vachon, Catherine W. Striley, Mollie R. Gordon, Miriam L. Schroeder, Emily C. Bihun, Jonathan M. Koller, Kevin J. Black

**Affiliations:** 1 College of Arts and Sciences, Washington University in St. Louis, University City, USA; 2 Department of Epidemiology, Colleges of Medicine and Public Health & Health Professions, University of Florida, Gainesville, USA; 3 Menninger Department of Psychiatry and Behavioral Sciences, Baylor College of Medicine, Houston, USA; 4 Private Practice, St. Louis, USA; 5 Department of Psychiatry, Washington University School of Medicine, St. Louis, USA; 6 Departments of Psychiatry, Neurology, Radiology, and Neuroscience, Washington University School of Medicine, St. Louis, USA

**Keywords:** Tourette syndrome, prevalence, epidemiology, method, video-audio media, video recording

## Abstract

Population-based assessment of Tourette syndrome (TS) and other tic disorders produces a paradox. On one hand, ideally diagnosis of tic disorders requires expert observation. In fact, diagnostic criteria for TS explicitly require expert assessment of tics for a definite diagnosis. On the other hand, large-scale population surveys with expert assessment of every subject are impracticable. True, several published studies have successfully used expert assessment to find tic prevalence in a representative population (e.g. all students in a school district). However, extending these studies to larger populations is daunting.

We created a multimedia tool to demonstrate tics to a lay audience, discuss their defining and common attributes, and address features that differentiate tics from other movements and vocalizations. A first version was modified to improve clarity and to include a more diverse group in terms of age and ethnicity. The result is a tool intended for epidemiological research. It may also provide additional benefits, such as more representative minority recruitment for other TS studies and increased community awareness of TS.

## Introduction

Some important questions in Tourette Syndrome (TS) require large-scale epidemiological studies. To give one example, studies have not yet had the power to definitively establish whether TS is equally common in people of African versus European descent. In the U.S., although diagnosis and treatment are about twice as common in European Americans ( CDC, 2009), three prior studies in the U.S., though limited in various ways, all found tics to be more common in minorities ( Costello *et al.*, 1996; Lapouse & Monk, 1964; [Table 4]; personal communication Costello EJ to KJB, 1999; personal communication Peterson BS to KJB, 2008; Peterson *et al.*, 2001). The results may differ so dramatically because of true genetic or epigenetic differences between racial groups ( Robertson *et al.*, 2009), or because social determinants of health care create barriers to diagnosis or treatment that create an artifactual difference in apparent prevalence ( American Psychiatric Association, 2013, under Tic Disorders/Culture-Related Diagnostic Issues; CDC, 2009; Olfson *et al.*, 2011). Settling this question will require large-scale prevalence studies that recruit an adequate, representative sample of minority populations.

Cubo (2012) reviews several factors that complicate epidemiological research on TS. One is that such studies generally must rely on assessments by lay interviewers. Although that approach has been very useful for psychiatric epidemiology in general, the validity may reasonably be questioned in the case of TS. There can be difficulties in conveying adequate descriptions of movements by words alone; probable miscategorization or failure to recognize some abnormal movements by both subjects and lay interviewers; the broad differential diagnosis of tics, including other movement disorders and normal movements; and misinterpretation of typical tics due to their intermittent nature, suppressibility and fluctuating severity over time or in response to the environment.

We were especially concerned that some respondents with tics, or whose children had tics, might not correctly interpret written descriptions of tics but would recognize the tics if they saw them. Supporting the potential importance of this concern, epidemiological studies that included expert examination ( Comings *et al.*, 1990; Cubo *et al.*, 2011; Hornsey *et al.*, 2001; Jin *et al.*, 2005; Khalifa & von Knorring, 2003; Khalifa & von Knorring, 2005; Kurlan *et al.*, 1994; Lanzi *et al.*, 2004; Mason *et al.*, 1998; Wang & Kuo, 2003) generally report a several-fold higher prevalence of tic disorders than do other epidemiological studies ( CDC, 2009; Scahill *et al.*, 2014).

To address these issues, we developed a multimedia screening interview to enhance population-based ascertainment of tic disorders by lay interviewers (“VISIT-TS”, Gordon *et al.*, 2010). A video presented and discussed typical tics, addressed a few difficulties in differential diagnosis, and then presented questions to gather the information required for diagnosis by DSM-IV-TR ( American Psychiatric Association, 2000) and DSM-5 ( American Psychiatric Association, 2013). After initial testing and application (unpublished report, Striley CW, Black KJ, Kelso N, and Vagelakos L), we revised the instrument. Here we describe the approach we took and the result: VISIT-TS v. 2.

## Methods

We first reviewed previous methods including the Yale Child Study Center questionnaire ( Findley *et al.*, 1999; Jagger *et al.*, 1982), the Kiddie SADS semi-structured interview (K-SADS-PL) ( Kaufman *et al.*, 1997) and the interviews used by Apter *et al.* (1993); Gillberg & Rasmussen (1982, Appendix); Hornsey *et al.* (2001) and Mason *et al.* (1998) who used the Apter questions and the National Hospital Interview Schedule for GTS ( Rickards & Robertson, 2003; Robertson & Eapen, 1996); Appendix I in Khalifa & von Knorring (2003); and Table 1 in Linazasoro *et al.* (2006). We also reviewed the Diagnostic Confidence Index ( Robertson *et al.*, 1999), the YGTSS ( Leckman *et al.*, 1989; Storch *et al.*, 2005), and the parent and child self-report forms used by the Tourette Syndrome Association International Consortium for Genetics (1999). An expert in psychiatric epidemiology (CWS) developed the questions that would be posed, in consultation with a movement-disorders-trained neuropsychiatrist (KJB). The interview was designed to address both current (past month) and lifetime symptoms and included information needed for TSSG, DSM-IV-TR and DSM-5 criteria for TS.

We wrote a script addressing the following aims: demonstrate tics, discuss their defining and common attributes, and address features that differentiate tics from other movements and vocalizations. We then selected video clips from patients and research volunteers who gave written permission to re-use their video separately from patient care or the research study they had participated in. We also obtained permission from people with tics to re-use selected video clips that they had already made publicly available on YouTube. The final video was produced by Ty Travis (San Tan Valley, Arizona, USA). We dubbed the final product VISIT-TS, for “Video-Integrated Screening Instrument for Tics and Tourette Syndrome” ( Gordon *et al.*, 2010).

The first version of VISIT-TS was used in an initial reliability and validity study that provided experience and initial feedback from interviewees and staff (unpublished report, Striley CW, Black KJ, Kelso N, and Vagelakos L). It was also shared with about a dozen other movement disorders experts and we reviewed their feedback. In response to this initial experience and feedback, we made many changes, including new video clips, thereby reducing the amount of time the narrator is shown and showing more diversity in ethnicity and age. We added and improved graphics, including written text while examples of tics appear in the background. To avoid confusion, we removed videos showing examples of non-tic movements, and we eliminated medical terms unfamiliar to the general public, such as chorea. Finally, we restored an unintentionally omitted question on lower facial tics. Here we describe the revised product, version 2 of VISIT-TS.

## Results

The revised VISIT-TS multimedia tool includes almost 100 video clips defining and demonstrating tics including simple and complex motor tics as well as simple and complex vocal tics, edited to a length of 5 minutes. Following the clips, 16 questions are presented in written and spoken form, one at a time, accompanied in most cases by brief video of the phenomenon being ascertained (see Appendix 1; question 4 of the video, at about 6:09, is a good brief demonstration). The questions take another 5 minutes. The video clips demonstrate adults and children in similar numbers, both sexes (male:female ≈ 5:4), and include some ethnic diversity (about 1 in 8 clips are Hispanic or non-white). VISIT-TS v. 2 is freely available for noncommercial use at https://zenodo.org/collection/user-kjb or at http://dx.doi.org/10.5281/zenodo.55604.

## Discussion

This approach is based on the premise that survey respondents will respond more accurately about tics in themselves (or their children) after the interviewer shows them a brief video about tics than they would if only asked about history by questionnaire or by cross-sectional lay observation. Because tics can come and go, can be suppressed, and often resemble intentional movement or vocalizations, diagnosis of tic disorders can be challenging ( Black *et al.*, 2016; Cubo, 2012).

Some data are available to judge the sensitivity of lay diagnostic instruments for tic disorders. In two studies, about half of the children who had previously been diagnosed with TS were missed by research screening: 1 of 2 in Landgren *et al.*, 1996 and 8 of 15 in Snider *et al.*, 2002. Conversely, routine clinical assessment for tics is also insensitive; Kadesjo & Gillberg (2000) report that a tic diagnosis had been considered during child psychiatric treatment in only 1 of 18 children with TS. Khalifa & von Knorring (2003) examined the sensitivity of their questionnaire but only by comparing questionnaire responses from parents to those from teachers. It appears Wang & Kuo (2003) collected physician examination data on questionnaire-negative children, but those data were not reported. Linazasoro *et al.* (2006) do not specify whether any of the tics diagnosed by a physician observing a classroom of students for 20 minutes were missed by parent or teacher questionnaires. Stefanoff *et al.* (2008) diagnosed a tic disorder in 6% of children whose parents and teachers noticed no tics; this is more remarkable given that the diagnosis rate in screen- *positive* children was only 18%. Cubo *et al.* (2011) found sensitivities of 36%–73% for questionnaires completed by teachers, observers or parents. In a recent study, a semistandardized diagnostic interview (the DISC) captured only about half the cases of TS, and there was little agreement between DISC results and expert clinician diagnosis ( Lewin *et al.*, 2014).

The most detailed data on the sensitivity of questionnaires for tic diagnosis come from the study of Mason *et al.* (1998). They gave questionnaires containing the 4 tic screening questions of Apter *et al.* (1993) to students, parents and teachers. They also screened for tics with direct classroom observation by Dr. Mason, a psychologist trained in tic detection at the Queen Square, London, TS center; she watched each classroom for an hour, 2 minutes per student. To confirm the diagnosis, Mason then directly examined all 16 consenting screen-positive students in a traditional clinical setting. Importantly, Mason also examined 8 students randomly chosen from screen negatives, i.e. those who had no tics reported by themselves, parents, or teachers, and no tics observed in the classroom. Remarkably, 3 of the 8 had at least one tic when examined directly, counted only if it had been present for at least a year by history! This very high rate of missed chronic tic disorders (37.5%) suggests that traditional questionnaires and interviews are insufficiently sensitive. VISIT-TS was designed to improve sensitivity by making sure subjects and parents have seen typical tics on video before answering questions about them.

Linazasoro & colleagues (2006) used a method somewhat similar to the VISIT-TS approach, *i.e.*, they showed a videotape of tics as part of an initial lecture to parents and teachers, followed by a survey that included a short written description of tics. Independently, “all children were directly observed in the classroom by an expert clinician in the field of tics who diagnosed tics based exclusively on the characteristics of the movements”, with a limit of 20 minutes’ observation per classroom (p. 2107). However, the authors note limitations of their work including the fact that children were observed collectively, for a relatively brief period of time, and while engaged in school work, when tics may have been suppressed. The questionnaires actually identified more children (98) than the expert (57), suggesting either that parents and teachers overdiagnosed some movements as tics, or that they were describing tics present in the past but no longer present, or that the classroom observation was not an adequately sensitive clinical comparison. A videotape demonstrating tics was released ( Tourette Syndrome Association, 1990), but it was intended for a professional audience rather than for epidemiological studies.

The VISIT-TS also has limitations. The DSM and TSSG criteria explicitly require application by properly trained experts, so VISIT-TS is primarily intended as a screening tool rather than as a substitute for clinical expertise. The sensitivity of VISIT-TS has not been reported. Furthermore, its specificity may be limited; the video includes only minimal information that could help distinguish tics from chorea, dystonia, stereotypies ( *sensu stricto*) or other abnormal movements, and after all such differential diagnosis generally requires clinical expertise. Nevertheless, a 5- to 10-minute video-illustrated questionnaire is probably a reasonable compromise for epidemiological or other tic studies that require screening large population samples for tic disorders. Finally, the video inquires separately about tics in different body locations, in hopes of improving sensitivity. However, since our primary intent was to identify presence or absence of tics, not to distinguish tics by body part affected, further experience with VISIT TS may allow shortening the video further by reducing the number of questions.

## Data availability

The data referenced by this article are under copyright with the following copyright statement: Copyright: © 2016 Vachon MJ et al.

The video can be found here: http://dx.doi.org/10.5281/zenodo.55604 ( Vachon *et al.*, 2016).

## Appendix 1

### Questions asked at the end of the VISIT-TS v. 2

1. Have you ever had eye movement tics?
2. Have you ever had repeated blinking or winking, like the tics in the video?
3. Have you ever had eyebrow tics?
4. Have you ever had mouth, tongue or jaw tics?
5. Does any part of your upper body, such as your head, shoulders, or arms jerk, turn or move unexpectedly or differently like in the video?
6. Has the middle or lower part of your body ever moved differently, like your stomach muscles contracting, your legs kicking or feet moving, over and over?
7. Have you sometimes repeated sounds over and over, like clicking, clucking, humming, grunting or smacking?
8. Have you sniffed or cleared your throat over and over, even when you didn’t have allergies or a cold and your nose wasn’t runny or itchy?
9. Have you had any repeated movements that are always done in the same way and involve more than one muscle group—like eyes *and* mouth, or shoulder plus arm? These tics may seem like they are being done on purpose or intentionally at times, but usually they are not.
10. Have you done things over and over like:
  a. Adjusting your clothes?
  b. Making obscene gestures?
  c. Patting yourself?
  d. Twisting your hair?
  e. Scratching your foot inside your shoe?
11. Have you sometimes repeated words over and over, or yelled out phrases over and over?
12. The video showed several behaviors, including making noises and movements, that people did over and over. Do you feel you have any tic-like behavior that has not already been mentioned? If yes, please describe it.
*if Yes to any previous question, continue:*
13. Did your “YES” answer, or answers, refer to something that started before your 18th birthday, that is when you were age 0 through 17 years old?
14. Did your “YES” answer, or answers, refer to something that happened many times a day most days for some period of time?
15. Did your “YES” answer, or answers, refer to something that kept happening over a period of a year or more?
*if Yes to question 15, continue:*
16. You said you had a year go by during which you had tics most of the time. During that year, did you ever have a time when all the tics were gone for 3 months straight?
