# Supplementary material for: VISIT-TS: A multimedia tool for population studies on tic disorders
Source: F1000Res. 2016 Oct 7;5:1518. Originally published 2016 Jun 27. [Version 2] doi: 10.12688/f1000research.7196.2 (PMC5089139; doi:10.12688/f1000research.7196.2)
Supplement: Peer review history

# Peer review: Referee response for version 2

**DOI:** 10.5256/f1000research.10427.r16869

**Published:** 2016-11-07

**Authors:** Shawn Smyth, James R. Brašić

**Affiliations:** 1 Parkinsons and Movement Disorders Center of Maryland, Johns Hopkins School of Medicine, Baltimore, MD, USA; 2 Sinai Hospital, Baltimore, MD, USA; 3 The Russell H. Morgan Department of Radiology and Radiological Science, Johns Hopkins University School of Medicine, Baltimore, MD, USA

Vachon and colleagues present a structured assessment designed to assist trained lay interviewers in assessing Tourette syndrome and other tic disorders in larger population studies. This tool is a well thought out instrument that will likely help fill a void in the area of the epidemiology of tic disorders. The use of trained lay interviewers to conduct structured psychiatric interviews has been used for population studies of children, adolescents, and adults with possible mental disorders. However, merely viewing a video may not be adequate to train people to distinguish movement disorders. Interrater reliability can verify the skills of a trained rater to identify the movements. However, the publication of this article can be justified by the dearth of trained individuals to screen large populations. This article may provide a tool to advance population studies around the world. We think only minor revisions might be needed. 

 The title is confusing as it employs an acronym which should either be defined within the title, or the title could be changed to not include the name of the instrument. Readers may be unfamiliar with the tool. Since the instrument has a long name, the title of the article could simply describe the procedure, e.g., “A strategy for conducting population studies of Tourette syndrome utilizing trained lay raters.”

 The abstract represents a suitable summary of the work and is adequate in its description of the study tool and design. Unlike articles presenting instruments along with psychometric properties (Goetz *et al.,* 2008), this article presents a tool without any properties, results of use, or other such data. Thus, the abstract can be enhanced by describing the limitations of this tool (i.e. lack of reliability and validity data) while still making it clear that the instrument is being offered to the research and lay communities given the lack of adequate tools for population studies on tic disorders.

 Regarding the article content, please define the acronyms. In particular, under Methods in the last line of the first paragraph, “TSSG” may be unfamiliar to readers. However, the design and methods are adequately explained and are appropriate for a publication which addresses the struggles of doing epidemiological studies on tic disorders. 

 It is less typical for a study to review the development of a tool without some preliminary data when applying the tool to the population. As F1000Research features quick and transparent publications which can then be openly reviewed and digested by the scientific community, we think it would be an optimal environment to include the unpublished preliminary reliability and validity data of the VISIT-TS (first version). This would be adequate to assist the readers of this journal to make some assessment of the suitability of this procedure for scientific research. Alternatively, the information about the unpublished reliability and validity studies conducted by the authors can be presented as a separate pilot study.

 The discussion could be improved by focusing a little more on the actual VISIT-TS and what is known about it, and less on the limitations of our current research methods and tools. Some of the latter information would be better incorporated into the introduction, which already reviews the current state of epidemiological research in tic disorders and makes a case for why the VISIT-TS is needed. Readers would also be very interested in the authors’ ideas regarding possible future research directions or studies that could be performed with this multimedia tool, such as:

- A group of lay individuals could be trained to use this strategy. Then the trained raters could be presented a series of new videotapes of people with and without tics. The findings could be compared and contrasted with the findings of expert raters.
- Populations from the community could be given tests about tics before and after viewing the video to assess whether the video is an effective tool to educate children, parents, teachers, and members of the general community how to identify tics.
- Evaluation of the reliability and validity of this procedure could be performed in multiple future studies with different populations.

Both the included video and list of questions are very helpful for the readers’ understanding regarding how this tool is administered and what the experience is like for the participants.

We have read this submission. We believe that we have an appropriate level of expertise to confirm that it is of an acceptable scientific standard.

## References

[1] GoetzCGTilleyBCShaftmanSRStebbinsGTFahnSMartinez-MartinPPoeweWSampaioCSternMBDodelRDuboisBHollowayRJankovicJKulisevskyJLangAELeesALeurgansSLeWittPANyenhuisDOlanowCWRascolOSchragATeresiJAvan HiltenJJLaPelleN: Movement Disorder Society-sponsored revision of the Unified Parkinson's Disease Rating Scale (MDS-UPDRS): scale presentation and clinimetric testing results. *Mov Disord*.2008;23(15) : 10.1002/mds.22340 2129-70 10.1002/mds.22340 19025984

# Peer review: Referee response for version 2

**DOI:** 10.5256/f1000research.10427.r16867

**Published:** 2016-10-31

**Authors:** David R. Shprecher

**Affiliations:** 1 Banner Sun Health Research Institute, Sun City, AZ, USA; 2 Department of Neurology, University of Utah, Salt Lake City, UT, USA

The authors carefully reviewed the epidemiology of tics and Tourette syndrome and explained the need for more sensitive, broadly applicable screening tools. To address this need, they have developed a 10-minute video that shows nearly 100 tic examples and then asks a brief screening questionnaire.

 The introductory section could benefit from some reference to the Avon study, which is considered landmark, as it was the only prospective, population-based study on prevalence of chronic tic disorders in a birth cohort (Scharf JM *et al,*. 2012).

 The video has already been improved with input from the first round of reviewers. In order to avoid overwhelming or confusing the layperson, non-tic hyperkinetic disorders are appropriately left out. The emphasis was appropriately on improving sensitivity, so that potential tic disorders can be identified and further screened by a researcher or clinician. However, there still was one instance where the video appears to have sacrificed sensitivity and waded into murky waters. This is where examples of repetitive behaviors that are not tics (because they are compulsions) were given. Repetitive hand washing was a great example. However repeating a movement a certain number of times “because it feels right” may not be an appropriate example. Compulsions are performed to relieve anxiety, however compulsive tics are performed in order to relieve an urge (which can include evening out or repetition.) There is still equipoise about how to classify compulsive tics, therefore; in order to maintain sensitivity of the instrument, this clip might best be modified to use a more classic example of a compulsion (i.e., checking that the lights are off or the door is locked, even though you’ve just checked.)

 There are a couple of concerns about the diagnostic utility of the questionnaire at the end of the video. Question #10 on the screening interview at the end of the video (“Have you ever done things over and over like:)”) gave 2 apt examples (“obscene gestures” and “patting yourself”) but there rest are habits that are probably not specific enough for tic disorders. These include “twisting your hair,” “scratching your foot inside your shoe” or “adjusting your clothes.”  Unless there is data to support these behaviors as being specific to tic disorders, the examples in this question might be appropriately replaced with behaviors that are more specific to tic disorders. Examples might include, “repeating words or phrases,”  “flicking your fingers” and  “poking or hitting yourself.”

 Another concern is that the questionnaire appears to be designed according to DSM-IV criteria, likely because the project was started before the DSM-V criteria for TS were finalized in 2013 (American Psychiatric Association, 2013 http://dsm.psychiatryonline.org/doi/10.1176/appi.books.9780890425596.dsm01). DSM-V removed the requirement that one year of tics be present consecutively during childhood (a total of one year must be present, but it does not need to be continuous, i.e. it can be separated over time). DSM-V also removed a 3-month tic free period as exclusion for the diagnosis of TS. This video appears to specifically ask about the 3-month tic free period. If it is going to be used to screen according to current DSM-V criteria, then this question should be removed. 

 The discussion aptly points out how poorly specific a brief interview with children can be for diagnosis of tics (with 37.5% being missed even after 2 hours of observation by an expert.) The discussion could be improved in two ways. First, it could point out an additional potential value of this video beyond screening the public for tics. Along these lines, it could be useful in training researchers and even their staff to conduct broad scale assessments of tics. For example, the Monroe County study at the University of Rochester (Kurlan R *et al,.* 2001), trained technicians using 25 different video examples of tics before they went on to conduct a total of 1596 interviews of school children. They found a much higher prevalence of chronic tics, TS, and tics NOS than other epidemiological studies using less sensitive methods have (18.5 % of children had at least some form of tic, and 7% were found to have TS). This is much higher than other epidemiological research, but used a more sensitive (yet less specific instrument). A second improvement to the discussion would be, following the limitations section, a conclusion that proposes next steps. For example, a validation study could be proposed. Additionally, further improvements to the instrument could be considered (in the future) to create educational tools aimed at improving diagnostic sensitivity of clinicians (and researchers) and raising awareness about tics in the general population (particularly minorities that are hard to reach, and may be under-diagnosed.)

I have read this submission. I believe that I have an appropriate level of expertise to confirm that it is of an acceptable scientific standard.

## References

[1] ScharfJMMillerLLMathewsCABen-ShlomoY: Prevalence of Tourette syndrome and chronic tics in the population-based Avon longitudinal study of parents and children cohort. *J Am Acad Child Adolesc Psychiatry*.2012;51(2) : 10.1016/j.jaac.2011.11.004 192-201.e5 10.1016/j.jaac.2011.11.004 22265365PMC3314954

[2] American Psychiatric Association: Neurodevelopmental Disorders.2013; 10.1176/appi.books.9780890425596.dsm01 10.1176/appi.books.9780890425596.dsm01

[3] KurlanRMcDermottMPDeeleyCComoPGBrowerCEapenSAndresenEMMillerB: Prevalence of tics in schoolchildren and association with placement in special education. *Neurology*.2001;57(8) :1383-8 1167357610.1212/wnl.57.8.1383

# Response

**Published:** 2016-10-31

**Authors:** Kevin J Black

**Affiliations:** Department of Psychiatry, Washington University in St Louis, USA

I am very appreciative of Dr. Shprecher's thoughtful comments.

- We envisioned VISIT-TS being used primarily for screening, a setting in which sensitivity was more of a concern than specificity, so we were trying to be inclusive. Still, in retrospect, I think overall I agree with the suggestions on compulsions and other repetitive behaviors.
- The comment about DSM-IV-TR is correct, but we chose to leave in the 3 questions about timing ("many times a day most days," "kept happening over a period of a year or more," and no 3-month period without tics). We argue that the 3-month question can simply be ignored for DSM-5, and my colleagues and I (at least) are still interested in DSM-IV-TR diagnoses. Nevertheless, to best fit DSM-5, which removed the "many times a day ... nearly every day" requirement, we probably should add a question like, "Was your first tic at least one year before your most recent tic?"
- Similarly, we may consider changing the age of onset question to ask for a(n approximate) age rather than a dichotomous yes or no regarding before age 18. My read of the science is that the age limit should probably be around age 21-25, and the TSSG criteria required age 21 rather than 18 [1].
- The comments on the introduction and discussion are also helpful.

Again, thanks.

 [1] The Tourette Syndrome Classification Study Group. Definitions and classification of tic disorders. *Arch Neurol*. 1993 Oct. 50(10):1013-6. [Medline].

# Peer review: Referee response for version 1

**DOI:** 10.5256/f1000research.7752.r14613

**Published:** 2016-07-12

**Authors:** Kerstin J. Plessen, Julie Hagstrøm

**Affiliations:** 1 Department of Clinical Medicine, Faculty of Health and Medical Sciences, University of Copenhagen, Copenhagen, Denmark; 2 Child and Adolescent Mental Health Centre, Mental Health Services - Capital Region of Denmark, Copenhagen, Denmark

In the article “VISIT-TS version 2: A multimedia tool for population studies on tic disorders” by Vachon, MJ, Striley, CW, Gordon, MR, Schroeder, ML., Bihun, EC, Koller, JM, and Black, KJ, a new method of classifying tics by lay persons is proposed as an aid in larger scale population studies.

 The authors provide a clear account for the need for improving existing methods of detecting and differentiating tics and present an interesting solution in the form of their new multimedia tool. It is a positive aspect that participants with tics include different age groups, races, and both sexes. However, some of the video clips present one separate tic, whereas several tics are present in the video.

 The rationale for developing the screening tool is its integration in large-scale epidemiological studies. Even though a few epidemiological studies focusing on tics and Tourette have been carried through, future methods will probably offer possibilities for screening cohorts for several co-occurring conditions. The authors may thus consider the possibility of further pruning the video (almost 10 minutes) and comparing two versions (e.g. the long one and a shorter version focusing on the different types of tics, which are more or less presented in the first few minutes of the video) for their validity against expert ratings. The shorter the time used for a screening, the more it will be used in big cohort studies. This could be integrated into future perspectives at the end of the manuscript.

 It would also be interesting to carry out similar tests of the sensitivity as mentioned in the article by comparing the VISTS-TS to clinician ratings.

 Storch et al. (2005) is used as a reference for the YGTSS – the authors might consider adding the reference of Leckman’s (1989) original work.

We have read this submission. We believe that we have an appropriate level of expertise to confirm that it is of an acceptable scientific standard.

# Response

**Published:** 2016-07-12

**Authors:** Kevin J Black

**Affiliations:** Department of Psychiatry, Washington University in St Louis, USA

We appreciate the thoughtful suggestions from Drs. Plessen and Hagstrøm.

 Our primary intent was to help lay people decide whether they (or their child) had any tics, not to distinguish which tics they had. I see from your response that we did not make that point clear. I think there are benefits to presenting and asking about different tics separately, even for our more limited goal, but your perspective also suggests an opportunity to shorten or otherwise improve on the video.

 You wrote, "some of the video clips present one separate tic, whereas several tics are present in the video." I believe you are saying that some video clips may show two tics while the narration is discussing only one of them. If I understood that correctly, it's a good point that we had not considered. We may be able to address that in a future version of the video.
